# A Potential Alternative against Neurodegenerative Diseases: Phytodrugs

**DOI:** 10.1155/2016/8378613

**Published:** 2016-01-06

**Authors:** Jesús Pérez-Hernández, Víctor Javier Zaldívar-Machorro, David Villanueva-Porras, Elisa Vega-Ávila, Anahí Chavarría

**Affiliations:** ^1^Departamento de Medicina Experimental, Facultad de Medicina, Universidad Nacional Autónoma de México, 06726 México, DF, Mexico; ^2^Posgrado en Biología Experimental, Universidad Autónoma Metropolitana-Iztapalapa, 09340 México, DF, Mexico; ^3^Programa de Becas Posdoctorales, Universidad Nacional Autónoma de México, 04510 México, DF, Mexico; ^4^Posgrado en Ciencias Biomédicas, Universidad Nacional Autónoma de México, 04510 México, DF, Mexico; ^5^División de Ciencias Biológicas y de la Salud, Universidad Autónoma Metropolitana, 09340 México, DF, Mexico

## Abstract

Neurodegenerative diseases (ND) primarily affect the neurons in the human brain secondary to oxidative stress and neuroinflammation. ND are more common and have a disproportionate impact on countries with longer life expectancies and represent the fourth highest source of overall disease burden in the high-income countries. A large majority of the medicinal plant compounds, such as polyphenols, alkaloids, and terpenes, have therapeutic properties. Polyphenols are the most common active compounds in herbs and vegetables consumed by man. The biological bioactivity of polyphenols against neurodegeneration is mainly due to its antioxidant, anti-inflammatory, and antiamyloidogenic effects. Multiple scientific studies support the use of herbal medicine in the treatment of ND; however, relevant aspects are still pending to explore such as metabolic analysis, pharmacokinetics, and brain bioavailability.

## 1. Introduction

Neurodegenerative diseases (ND) such as Alzheimer's (AD) and Parkinson's disease (PD) and multiple sclerosis (MS) primarily affect the neurons in the human brain and are characterized by deterioration of neurons or myelin sheath, sensory information transmission disruption, movement control, and more [1]. The greatest risk factor for ND is aging, which carries mitochondrial dysfunction, chronic immune-inflammatory response, and oxidative stress [2, 3], the major causes of neuronal damage and death. Nowadays, ND are chronic and incurable conditions whose disabling effects may continue for years or even decades representing an enormous disease load, regarding human suffering and economic cost. The ND are more common and have a disproportionate impact on countries with longer life expectancies and represent the fourth highest source of overall disease burden in the high-income countries. According to the World Health Organization, 37 million people currently have dementia worldwide, and about 50% of them are being affected by AD and this number is expected to grow up to 115.4 million people by 2050 [4].

**Figure 1:**
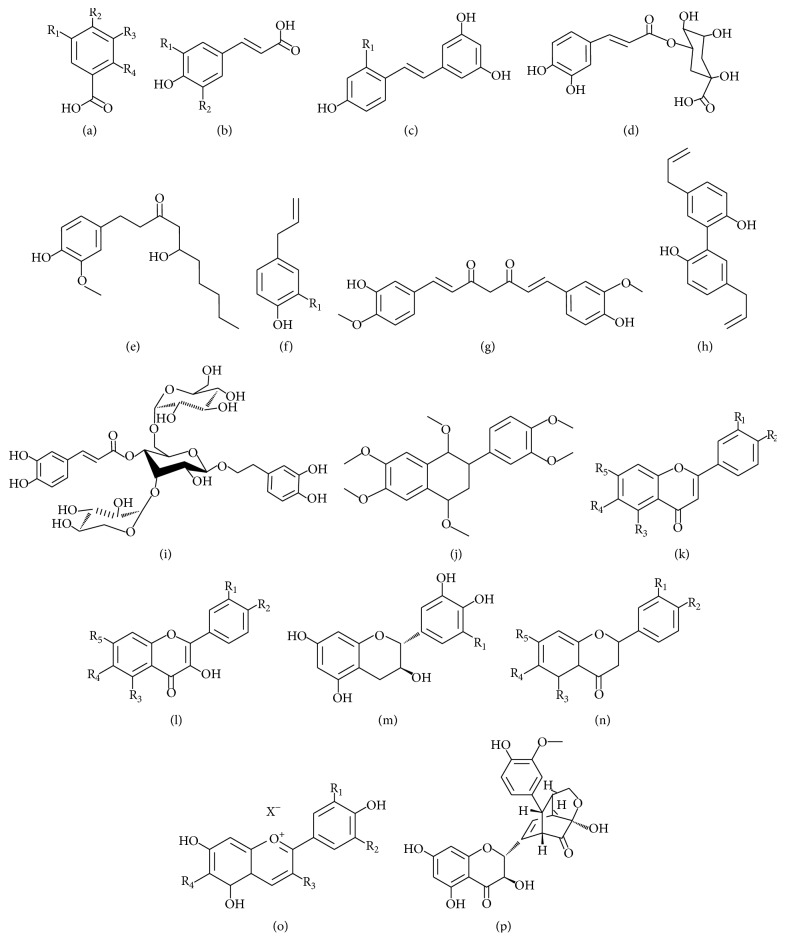
Representative polyphenol compounds. (a) Benzoic acids: *p*-hydroxybenzoic acid R_1_ = R_3_ = R_4_ = H, R_2_ = OH; protocatechuic acid R_1_ = R_4_ = H, R_2_ = R_3_ = OH; gallic acid R_1_ = R_2_ = R_3_ = OH, R_4_ = H; and salicylic acid R_1_ = R_2_ = R_3_ = H, R_4_ = OH. (b) Hydroxycinnamic acids: coumaric acid R_1_ = R_2_ = H; caffeic acid R_1_ = OH, R_2_ = H; ferulic acid R_1_ = OMe, R_2_ = H; and sinapic acid R_1_ = R_2_ = OMe. (c) Stilbenes: resveratrol R_1_ = H; oxyresveratrol R_1_ = OH. (d) Hydroxycinnamoyl ester: chlorogenic acid. (e) Hydroxycinnamoyl derivatives: gingerol; (f) Chavicol R_1_ = H; eugenol R_1_ = OMe; (g) curcumin; (h) magnolol; and (i) echinacoside. Flavonoid compounds. (j) Nobiletin; (k) Flavones: apigenin R_1_ = R_4_ = H, R_2_ = R_3_ = R_5_ = OH; baicalein R_1_ = R_2_ = H, R_3_ = R_4_ = R_5_ = OH; chrysin R_1_ = R_2_ = R_4_ = H, R_3_ = R_5_ = OH; and luteolin R_4_ = H, R_1_ = OMe, R_2_ = R_3_ = R_5_ = OH. (l) Flavonols: kaempferol R_1_ = R_4_ = H, R_2_ = R_3_ = R_5_ = OH; quercetin R_4_ = H, R_1_ = R_2_ = R_3_ = R_5_ = OH. (m) Flavanols (+)-catechin R_1_ = H; (+)-gallocatechin R_1_ = OH. (n) Flavanones: hesperetin R_4_ = H, R_1_ = R_3_ = R_5_ = OH, R_2_ = OMe; naringenin R_1_ = R_4_ = H, R_2_ = R_3_ = R_5_ = OH; pinocembrin R_1_ = R_2_ = R_4_ = H, R_3_ = R_5_ = OH. (o) Anthocyanins: aurantinidin R_1_ = R_2_ = H, R_3_ = R_4_ = OH; cyanidin R_2_ = R_4_ = H, R_1_ = R_3_ = OH; pelargonidin R_1_ = R_3_ = R_4_ = H, R_2_ = OH; and peonidin R_2_ = R_4_ = H, R_1_ = OMe, R_3_ = OH. (p) Flavonolignans: silydianin.

**Figure 2:**
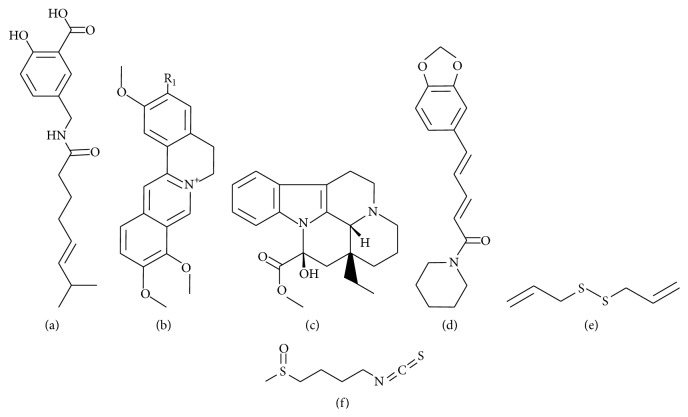
Some alkaloid compounds in plants. (a) Capsaicin; (b) protoberberines: jatrorrhizine R_1_ = OH, palmatine R_1_ = OMe; (c) vincamine; (d) piperine; (e) diallyl sulfide; and (f) sulphoraphane.

**Figure 3:**
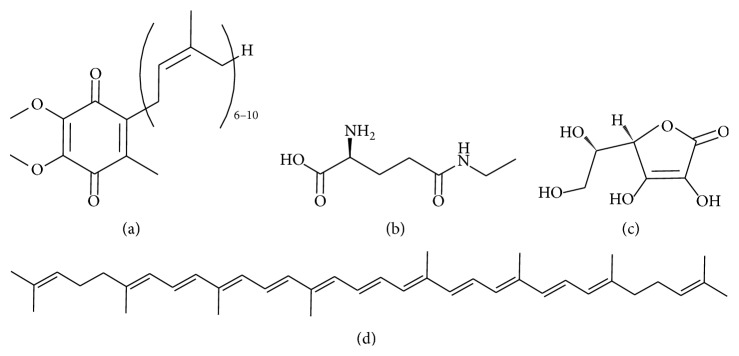
Some miscellaneous antioxidant compounds from plants. (a) Coenzyme Q_6–10_; (b) l-theanine; (c) ascorbic acid; and (d) lycopene.

Recently, a great number of natural medicinal plants have been tested for their therapeutic properties, showing that the raw extracts or isolated pure compounds from them have more effective properties than the whole plant as an alternative for the treatment of ND. These properties are due mainly to the presence of polyphenols (Figure 1), alkaloids (Figure 2), and terpenes (Figure 3(d)), among others, that are micronutrients produced by plants as secondary metabolites [5, 6]. There is substantial evidence (epidemiological studies, animal studies, and human clinical trials) that indicates that polyphenols reduce a wide range of pathologies associated with inflammation [7–9]. The main mechanisms of polyphenols include their well-characterized antioxidant effects [10, 11], inhibition of intracellular kinases activity [12], binding to cell surface receptors [13], and modifying cell membrane functions [14]. Also, recently the neuroprotective effects of polyphenols have been described in several models of ND and involve mainly signaling pathways mediators [15], modulation of enzymes in neurotransmission [16, 17], inhibition of neurotoxicity via ionotropic glutamate receptors [18], antiamyloidogenic [19], and anti-inflammatory effects [20]. This review focuses on the plant extracts or compounds isolated from plants that may hold potential in the treatment of the principal ND.

## 2. Etiology of Neurodegenerative Diseases

ND are incurable and disabling conditions secondary to progressive neuronal loss, which leads to chronic brain damage and neurodegeneration. The etiology of ND is still unknown, although several ND animal models showed associated damage with the blood-brain barrier, protein aggregation, toxin exposure, and mitochondrial dysfunction, which lead to oxidative stress and inflammation, and consequently neuronal death [21].

The blood-brain barrier controls the internal environment of the vertebrate CNS and represents the border between the capillary and the extracellular fluid of CNS neurons and glial cells; it also ensures specific brain homeostasis allowing adequate neuronal function [22]. Neurovascular changes normally occur as part of aging, but these are more evident in chronic ND [23]. About 20% of blood flow decreases in the aged brain, which associates with reduced protein synthesis [24]. Interestingly, this blood flow reduction is higher in the presence of any ND, which may lead to changes in intracellular pH and accumulation of interstitial lactate and glutamate [23, 25]. These changes are observed in specific brain regions in diseases such as AD, PD, MS among other CNS disorders [25–28].

Abnormal protein aggregation of specific regions and neuronal populations is a common feature among ND. For example, the *α*-synuclein inclusions in dopaminergic neurons from the substantia nigra are the main histopathological marker in PD [29]. Also, insoluble aggregates of the amyloid beta-peptide (A*β*) and neurofibrils composed of Tau protein are found in AD [30, 31] and hyperphosphorylated Tau aggregation in demyelination areas in MS [32]. Finally, superoxide dismutase 1 (SOD1) aggregations are present in amyotrophic lateral sclerosis (ALS) [33]. The main relevance of protein aggregates is that they lead to mitochondrial dysfunction inducing apoptotic neuronal death.

Redox state imbalance and chronic inflammation, a major cause of cell damage and death, characterize ND [34]. Reactive oxygen species (ROS) are key mediators of cell survival, proliferation, differentiation, and apoptosis [35, 36]. Excessive production of ROS by mitochondria and NADPH oxidase in oxidative stress is usually thought to be responsible for tissue damage associated with inflammation and ND [34, 36–38]. Moreover, many of the well-known inflammatory target proteins, including matrix metalloproteinase-9, cytosolic phospholipase A_2_, cyclooxygenase-2, inducible nitric oxide synthase (iNOS), and adhesion molecules, are associated with oxidative stress and induced by proinflammatory factors such as cytokines, peptides, and peroxidants agents [36, 39, 40]. Several studies have shown that ROS act as a critical signaling molecule to trigger inflammatory responses in CNS through the activation of the redox-sensitive transcription factors, including nuclear factor-*κ*B (NF-*κ*B) and activator protein-1 [34, 36–39].

Mitochondrial damage leads to neuronal oxidative damage in ND pathogenesis. ROS and reactive nitrogen species, which are normal byproducts of mitochondrial respiratory chain activity, are mediated by mitochondrial antioxidants such as manganese superoxide dismutase and glutathione peroxidase. In addition to the ROS generation, mitochondria are also involved with life-sustaining functions including adenosine triphosphate synthesis by oxidative phosphorylation, apoptosis, calcium homeostasis, mitochondrial fission and fusion, lipid concentration of the mitochondrial membranes, and the mitochondrial permeability transition. Mitochondrial disease leading to neurodegeneration is likely, at least on some level, to involve all of these functions [41]. In ND several mitochondrial alterations are found like bioenergetics anomalies in the process of oxidative phosphorylation and ATP production, defects of mitochondrial dynamics, increase sensitivity to apoptosis, and accumulation of damaged mitochondria with unstable mitochondrial DNA [2].

The proteins aggregation also plays an important role in mitochondrial dysfunction; for example, the accumulation of mitochondrial A*β* aggregates has been observed both in patients and in transgenic models of AD [42–44]. Additionally, inhibition of mitochondrial complex I occurs in PD patients [45] and the two principal models used for the study of PD. Rotenone—a natural compound used as an insecticide, piscicide, and pesticide—and 1-methyl-4-phenyl-1,2,3,6-tetrahydropyridine (MPTP)—a neurotoxin precursor of 1-methyl-4-phenylpyridinium (MPP^+^), which destroys dopaminergic neurons in the substantia nigra—both act by inhibiting mitochondrial complex I [21]. In ALS, mitochondrial SOD1 enzyme aggregates cause loss of mitochondrial function and induce cellular death by apoptosis [46]. This phenomenon is present in almost all ND and associated with inflammation, which is one of the points of therapeutic interest and study.

The CNS inflammation is dependent on inflammatory mediators produced mainly by glial cells, specifically microglia and CNS macrophages [47]. Microglial activation is crucial in the pathogenesis and the course of PD [48], AD [49], prion disease [50], and MS [51], among others. Uncontrolled microglia activation produces neuronal damage due to overproduction of proinflammatory mediators such as tumor necrosis factor *α* (TNF*α*) [52], and nitric oxide (NO), leading to the generation of oxidative stress and apoptotic cell death [48, 52, 53].

## 3. Main Therapeutic Effects of Plant Extracts

The plant extracts have become interesting candidates as therapeutic agents due to their antioxidant, anti-inflammatory properties, and chemical characteristics derived as follows.

*(i) Direct Uptake of Free Radicals*. Primarily polyphenols (Figure 1) and alkaloids (Figure 2) function as scavengers due to their multiple phenolic hydroxyl and nitrogen groups, respectively, which act as an electron donor to the aromatic ring. These systems are excellent nucleophiles that readily lose electrons and easily oxidize. Therefore, they can catch free radicals and react with ROS, such as superoxide, peroxyl, hydroxyl radicals, NO, nitrogen dioxide, peroxynitrite, and singlet oxygen [54–56].

*(ii) Chelation of the Divalent Cations in Fenton Reactions Involved*. Many polyphenol compounds chelate iron cations due to multiple hydrophilic groups and are efficient scavengers because phenolic groups inhibit iron-mediated oxyradical formation like other iron chelators, such as desferrioxamine, 1,10-phenanthroline, and pyridoxal isonicotinoyl hydrazone [57, 58].

*(iii) Modulation of Enzymes Associated with Oxidative Stress*. ND associate with molecular alterations in cell-signaling pathways that regulate cell proliferation and differentiation, such as the family of mitogen-activated protein kinases (MAPK). Abnormal activation or silencing of the MAPK pathway or its downstream transcription factors can result in uncontrolled cell growth leading to malignant transformation. Some plant compounds “switch on” or “turn off” the specific signaling molecule(s), depending on the nature of the signaling cascade they target, preventing abnormal cell proliferation and growth [59, 60].

## 4. Antioxidant and Anti-Inflammatory Properties in Central Nervous System

Flavonoids, a type of polyphenolic compounds found in fruits, vegetables, red wine, and green tea, reduce the risk to developing ND [61]. In 2010, Vuong and colleagues showed that cranberry juice in neuronal cultures significantly increased the activity of antioxidant enzymes such as catalase and SOD1 and protected neurons against H_2_O_2_ induced cell death, possibly due to the activation of survival pathways dependent from p38 and blocking death pathway associated with MEK1/2 and ERK1/2 [62]. A comparative study of two extracts of* Salvia* species,* S. hydrangea* and* S. macilenta*, also showed strong antioxidant properties, also at high concentrations (≥50 *μ*g/mL) they can inhibit DNA damage by free radicals. Moreover, these species not only showed no cytotoxic effect in cultured PC12 cells, a cell line derived from a pheochromocytoma obtained from rat adrenal medulla differentiated with neural growth factor, but also protected them from peroxide-induced cell death [63]. Similarly selaginellin, a compound extracted from the plant* Saussurea pulvinata*, showed a neuroprotective effect in a glutamate neurotoxicity model in PC12 cells by trapping ROS and regulating the expression of the* klotho* gene, which has an antiapoptotic role [64].

Ginger, the root of* Zingiber officinale*, an important specie used in the Chinese, Ayurvedic, and Tibia-Unani traditional medicine, has anti-inflammatory [65–67] and antioxidant [68] properties, among others. The hexane fraction of ginger extract and the methanol extract of* Ficus religiosa* sheet significantly decreased the production of NO, prostaglandin E2, IL-1*β*, IL-6, and TNF*α* through the inhibition of MAPK and NF-*κ*B in BV2 microglial cell line stimulated with lipopolysaccharide (LPS) [69, 70].

Similarly, the ethanol extract of* Knema laurina* exerted anti-inflammatory and neuroprotective effects in a BV2 microglial cell culture line, in HT-22 hippocampal neurons and in organotypic hippocampal cultures.* Knema laurina* reduced microglial production of NO and IL-6 through the inhibition of ERK1/2 and IKK*β* phosphorylation, and the subsequent translocation NF-*κβ* in microglial cells [71].

## 5. Therapeutic Opportunities for Plant Extracts in Central Nervous System Age-Related Changes

It is clear that aging is a critical factor for developing ND and facilitates the microglial promoted proinflammatory environment [72–74] and oxidative stress [75]. Therefore, studying potential drugs that prevent or retard age-related changes has become crucial. Natural antioxidants such as some cocoa derivatives have shown to contain higher flavonoids levels [76]. For example, acticoa, a cocoa-derived polyphenol extract, administered daily orally at 24 mg/kg dose in Wistar rats 15 to 27 months old, improved cognitive performance, increased life expectancy, and preserved free dopamine levels in urine [77]. Another extract with high antioxidant activity is silymarin, a standardized mixture of flavonolignans (Figure 1(p)) extracted from the* Silybum marianum* fruits and seeds [78]. The treatment with 400 mg/kg/day of silymarin during three days increased reduced glutathione (GSH) and SOD activity in the brain of aged rats [79]. Vincamine (Figure 2(c)), a monoterpenoid indole alkaloid purified from the* Vinca minor* plant, has antioxidant activity similar to vitamin E. This compound increased cerebral blood flow, glucose, and oxygen utilization in neural tissue and promoted the rise of dopamine, serotonin, and noradrenaline levels [80]. Also, the treatment of rats with vincamine during 14 days at a daily dose of 15 mg/kg reduced about 50% the brain iron levels, which suggests a beneficial effect in reducing the oxidative stress associated with the iron deposition in ND [81]. Moreover, paeonol, a compound extracted from the* Paeonia suffruticosa* cortex or* Paeonia lactiflora* root, has been ascribed to anti-inflammatory and antioxidant properties. Paeonol effects were tested in a model of neurotoxicity induced with D-galactose injected subcutaneously in aged mice. Paeonol prevented memory loss in this model since it increased acetylcholine and GSH levels and decreased the activity of acetylcholinesterase (AChE) and SOD1 in the hippocampus and cortex, positioning it as a potential drug useful in age-related ND [15]. Also,* Magnolia officinalis* compounds, magnolol (Figure 1(h)) and their isomer honokiol, were tested in a senescence-accelerated prone mice; this compound prevented learning and memory deterioration, as well as acetylcholine deficiency by preserving forebrain cholinergic neurons [18, 82].

## 6. Plant Compounds Used for Alzheimer's Disease

AD manifests as a progressive cognitive and behavioral disorder and is characterized by an immediate loss of memory secondary to neuronal loss in the limbic and association cortices. This neuronal death results from oxidative stress, neuroinflammation, and abnormal protein deposition [83], leading to a therapeutic opportunity for medicinal plants, which improve AD course principally by modulating A aggregation, AChE activity, oxidative stress, and inflammatory response [84].

Cryptotanshinone is an active component of* Salvia miltiorrhiza* with anti-inflammatory, antioxidant, and antiapoptotic properties [85, 86]. This compound crossed the blood brain barrier and decreased cognitive deficits in male IRC mice with scopolamine-induced cognitive impairments [87]. This compound also provided beneficial effects in patients with ischemia and cerebral infarct [88]. Additionally, cryptotanshinone reduced the A*β* aggregation in brain tissue and improved spatial learning and memory in APP/PS1 transgenic mice by promoting amyloid precursor protein metabolism via *α*-secretase pathway [89]. Silymarin also showed antiamyloid properties* in vitro*, and its chronic administration (half a year) significantly reduced the A*β* plaque burden associated with microglial activation, A*β* oligomer formation, and hyperactivity and disturbed behavior in APP transgenic mice [90]. The protective effect of silymarin on A*β* accumulation is attributable to the blockade of its aggregation, not to *β*-secretase inhibition [89]. The use of* Centella asiatica* in a dementia model in PSAPP mice improved memory retention in rodents [91, 92] and decreased amyloid deposition and the spontaneously A*β* plaque formation [93]. Likewise, the grape seed polyphenolic extract from* Vitis vinifera* attenuated the cognitive impairment observed in aging AD transgenic mice and decreased A*β* plaques deposition in the brains [94]. Nobiletin (Figure 1(j)), a flavonoid purified from* Citrus depressa* plant, prevents memory loss in APP695 transgenic mice and A*β* treated rats. This compound reduces the A*β* plaques amount in the hippocampus [95, 96], probably by reducing the inhibition of protein kinase A and cAMP response element-binding protein phosphorylation signaling cascade [97]. Nobiletin also stimulated long-term potentiation in organotypic hippocampal cultures [98]. Other compounds that can prevent A*β* aggregation by inhibition of the metabolic pathway that generates A*β* plaques are berberine, palmatine, jateorrhizine, epiberberine, coptisine, groenlandicine, and magnoflorine, alkaloids isolated from* Coptis chinensis* rhizome [99]. These compounds also exhibit AChE inhibiting properties [100, 101] and antidepressant effects [59] and enhance cognitive improvements [102]. Also, jateorrhizine (Figure 2(b)) and groenlandicine have significant peroxynitrite scavenging activities, while coptisine and groenlandicine present moderate total ROS inhibitory activities [99].

The ethanol extract from* Cassia obtusifolia* has potential use in AD, which reduced scopolamine-induced memory loss in mice by inhibiting AChE [103]. Similarly, methoxsalen, the main component of the aqueous extract of* Poncirus trifoliata*, inhibited AChE activity reducing memory loss and learning problems associated with a neurotoxicity* in vivo* model induced with trimethyltin [16]. In the AD model induced with ethylcholine aziridinium, which mimics the cholinergic hypofunction present in AD [104], piperine (Figure 2(d)), an alkaloid present in* Piper longum*, lowered the cognitive deficits and the hippocampal neurodegeneration associated with this AD model [105]. These effects could be probable due to its anti-inflammatory [106] and antioxidant activities [71].

The treatment for 5 weeks with L-theanine (Figure 3(b)), an amino acid present in green tea* Camellia sinensis*, significantly decreased memory loss associated with intraventricular A*β*_1–42_ AD model. L-theanine as well reduced cortical and hippocampal neuronal death, also inhibited lipid peroxidation and protein damage, and increased GSH levels, suggesting its potential use in AD prevention and treatment [17]. Also,* Dioscorea opposita* chloroform extract, which has been used to treat memory-related diseases such as AD and others ND, prevented neuronal death, and significantly increased spatial learning and memory improvement, probably due to its antiexcitotoxic and antioxidant effects [107].

Sanmjuanhwan (Sjh), a multiherbal formula from oriental traditional medicine, composed of* Morus alba*,* Lycium chinense*, and* Atractylodes japonica*, showed neuroprotective effects on primary neuronal cultures exposed to A*β*_25–35_. Sjh increased the expression of antiapoptotic proteins such as Bcl-2 and avoided cytochrome c release and caspase-3 activation [108].* B. monnieri* and its active components bacoside A, bacopaside I and II, and bacosaponin C [109, 110] have anti-inflammatory, antimicrobial, and antidepressant effects [111–113]. Treatment with* B. monnieri* prevented neuronal death by the inhibition of AChE activity in primary cortical culture pretreated with A*β*_25–35_ [114]. Furthermore, animals and volunteers treated with this plant presented enhanced memory [115–117]. The antioxidant effect of* S*-allyl cysteine (SAC), an amino acid isolated from aged garlic, was tested in the A*β*_25–35_-AD rat model, showing ROS scavenger activity* in vivo* [118]. Also, in the mice AD dementia model induced with the intracerebroventricular streptozotocin infusion, SAC pretreatment decreased p53 expression, restored Bcl-2 protein expression, reduced, and prevented DNA fragmentation [119].

Mono- and diacetyled cyanidin and peonidin, the purple sweet potato anthocyanins (PSPA; Figure 1(o)) extracted from* Ipomoea batatas*, can easily attract ROS, which has high clinical value as antioxidant therapy in AD and other ND [120, 121]. For example, pretreatment of PC12 cells with PSPA reduced A*β* toxicity preventing lipid peroxidation, caspase-3 activation, and A*β*-induced apoptosis, suggesting a possible use in the treatment of AD [122].

Finally, the use of ginseng,* Panax ginseng*, was evaluated in AD patients, those who received a daily dose 9 g of Korean red ginseng for 12 weeks showed a significant improvement in the AD assessment scale and the clinical dementia rating scale compared to control patients [123].

## 7. Plant Compounds for Parkinson's Disease Treatment

PD is the second most frequent ND and is primarily a movement disorder characterized by the loss of dopamine-producing neurons in substantia nigra. Activation of neuronal death pathways involves oxidative stress, neuroinflammation, and mitochondrial dysfunction [124].

Green tea extract and its isolated (–)-epigallocatechin-3-gallate polyphenol, as well as ginseng extract, have neuroprotective effects since their use diminished dopaminergic neuron loss in the substantia nigra and oxidative damage in an MPTP and its toxic metabolite MPP^+^ in PD animal models [125, 126]. Also,* Chrysanthemum morifolium*, which has antioxidant activity [126], inhibited MPTP-induced cytotoxicity and maintained cell viability of SH-SY5Y cell line, preventing ROS formation, decreasing Bax/Bcl2 ratio and caspase-3 activation [127]. The administration of 20 mg/kg of echinoside, a compound isolated from* Cistanche salsa*, before MPTP intoxication maintained striatal dopamine levels, reduced cell death, significantly increased the tyrosine hydroxylase enzyme expression, and reduced the activation of caspase-3 and caspase-8 expression, thus preventing neuronal death [128]. Likewise, silymarin treatment preserved dopamine levels, diminished the number of apoptotic cells, and preserved dopaminergic neurons in the substantia nigra of MPTP- and 6-hydroxydopamine-intoxicated mice (6-OHDA) [74, 129–131]. Besides, pelargonidin (Figure 1(o)), an anthocyanidin with neuroprotective effects, reduced the motor deficit and histological damage and prevented lipid peroxidation in the 6-OHDA model [132–134].

In the MPTP-intoxicated model of PD, SAC prevented lipid peroxidation and mitochondrial dysfunction [135], protected the striatum of mice from the morphological alterations with a reduction in TNF-*α* and iNOS expressions, and further reduction in astrocyte activation [136] and also, at 120 mg/kg dose by five days, partially ameliorated the MPTP-induced striatal and nigral dopamine and tyrosine hydroxylase depletion, attenuated the loss of manganese-dependent superoxide dismutase and heme oxygenase-1 activities, and preserved the protein content of these enzymes [137]. These findings suggest that SAC can exert neuroprotection since the origin of the dopaminergic lesion—at the substantia nigra—not only by using direct antioxidant actions but also through Nrf2 nuclear transactivation and phase 2 enzymes upregulation [137].

The commercial extract of* Anemopaegma mirandum*, a Brazilian tree, and the crude extract of* Valeriana officinalis* increased the viability of SH-SY5Y cells after rotenone exposure [138, 139], while the extract of* Rhus verniciflua* decreased ROS production, preserved the mitochondrial integrity, and decreased the number of apoptotic cells [140]. An extract from* Tripterygium regelii*, a plant with antioxidant properties, reduced oxidative stress-induced cell death through the inhibition of apoptotic cascades, preserved mitochondrial function, and promoted tyrosine hydroxylase expression and brain-derived neurotrophic factor (BDNF) production in H_2_O_2_ treated SH-SY5Y cells [141]. Also, in the MPP^+^-intoxicated SH-SY5Y cells, the orchid increased cell viability, decreased cytotoxicity and ROS production, and prevented caspase-3 activation by diminishing the Bax/Bcl2 ratio [142].

In the same model, the flavonoid luteolin (Figure 1(k))—a compound present in celery, green pepper, pear leaves, and chamomile tea—provided neuroprotection against oxidative stress [143]. Also, luteolin inhibited LPS induced microglial activation, as well as the production of TNF*α*, NO, and superoxide in a midbrain mixed primary cultures [144]. Pedicularoside A, a glycosylated phenylethanoid isolated from* Buddleja lindleyana*, has anti-inflammatory properties and is a good scavenger of superoxide anions and hydroxyl radicals [145]; it protected against MPP^+^-induced death in mixed midbrain primary culture by increasing tyrosine hydroxylase expression and decreasing caspase-3 cleavage [146]. The plant extract from* Uncaria rhynchophylla* decreased cell death and ROS production and increased GSH levels in cultured PC12 cells, while 6-OHDA-induced caspase-3 activation was attenuated preventing cell death and rotational behavior was significantly reduced in the 6-OHDA PD model [147]. The ethyl extract from* Myracrodruon urundeuva* displayed similar properties in mesencephalic cultured cells since it preserved cell viability and attenuated oxidative stress after 6-OHDA exposure [148].

*Panax notoginseng* (PN) has the property to increase the expression of certain molecules such as nestin and BDNF, promoting neural plasticity and recovery after cerebral ischemia [149, 150]. Also, PN induces the expression of thioredoxin-1, an oxidoreductase with antiapoptotic and cell growth promoter effects [151], reducing MPTP-induced cell death in PC12 cells [152]. Likewise, the root extract of* Withania somnifera* promoted axon and dendrite growth [153, 154] and also increased the levels of SOD1, catalase, and GSH, preventing deficit motor in MPTP-intoxicated animals [155].

The isoflavones daidzin, daidzein, and genistein contained in* Pueraria thomsonii* protected PC12 cells stimulated with 6-OHDA through the inhibition of the caspase-3 activation [156]. Moreover, genistein, a soy phytoestrogen, protected neurons from substantia nigra pars compact and attenuated the rotational behavior in a hemiparkinsonian 6-OHDA model [157]. Interestingly, the administration of* Mucuna pruriens* preceding 6-OHDA intoxication was more efficient than levodopa in controlling motor symptoms, since it restored dopamine and norepinephrine levels in the nigrostriatal track exhibiting a neuroprotective effect [158]. The mechanism of action of* Mucuna pruriens* is not fully understood; however, it has been proposed that increases the mitochondrial complex I activity without affecting the monoamine oxidase B activity, probably due to its high content of NADH and Q-10 coenzyme (Figure 3(a)), and its ability to scavenge ROS [159].

The herbal mixture Toki To (TKT), prepared of ten different plants (*Angelicae Radix*,* Pinelliae Tuber*,* Cinnamomi Cortex*,* Ginseng Radix*,* Magnoliae Cortex*,* Paeoniae Radix*,* Astragali Radix*,* Zanthoxyli fructus*,* Zingiberis siccatum Rhizoma*, and* Glycyrrhizae Radix*), has excellent results against PD [159]. TKT orally administered reduced motor symptoms such as bradykinesia, prevented dopaminergic neurons loss in the substantia nigra, and increased tyrosine hydroxylase and dopamine transporter expression in MPTP-intoxicated mice [159]. Through microarray it was determined that TKT* per se* regulates the expression of serum- and glucocorticoid regulated kinase gene (*sgk*), which are implicated in the PD pathogenesis [159].

*Psoralea corylifolia* seeds, specifically Δ3,2-hydroxybakuchiol monoterpene, which has been used for years in Chinese medicine for the treatment of cerebral aging and dementia [102, 160], protected SK-N-SH cells from MPP^+^ intoxication and prevented the dopaminergic neurons loss in MPTP-intoxicated mice by inhibition of the monoamine transporter [161, 162]. Also it is worth mentioning that* Rosmarinus officinalis*, a plant used as flavoring in Mediterranean cuisine, has antioxidant properties [163].* Rosmarinus officinalis* inhibits NO production [164] and protects dopaminergic neurons in different degenerative disease models [165–168], probably due to its a high content of polyphenols and terpenes such as carnosol, carnosic acid, and rosmarinic acid and antiapoptotic effects [169].

## 8. Plant Compounds for Cerebral Ischemia Management

In cerebral ischemia, severe neuronal damage occurs during the reperfusion period due to excitotoxicity, which consists of an overstimulation of* N*-methyl-d-aspartate (NMDA) receptors leading to glutamate production, which in turn triggers oxidative and inflammatory processes [26]. The intraperitoneal administration of 200 mg/kg of cactus polysaccharides, the active component isolated from* Opuntia dillenii*, prior to the middle cerebral artery occlusion showed neuroprotective effects [170, 171].* Opuntia dillenii* significantly reduced infarct volume, decreased neuronal loss in the cerebral cortex, and diminished importantly the nitric oxide synthase (NOS) synthesis, which is usually induced during the experimental period of reperfusion and ischemia [171]. Also, oral pretreatment with 30 and 50 mg/kg daily of* Smilacis chinae* rhizome (SCR) methanol extract reduced the histological changes associated with ischemic injury [172]. It is possible that SCR prevented excitotoxicity-induced neuronal death by decreasing ROS generation, similar to the observations made* in vitro* in primary cultures of cortical cells treated with 1 mM NMDA [172]. Additionally, intravenous pretreatment with silymarin reduced infarcted area size, as well as neurological deficits associated with ischemic damage [173]. Also, silymarin inhibited protein expression associated with inflammation such as iNOS, cyclooxygenase-2, myeloperoxidase, the nuclear transcription factor NF-*κ*B, and proinflammatory cytokines like IL-1*β* and TNF*α*, avoiding neurodegeneration associated with ischemia [173]. Similarly, SAC administration reduced infarct volume in a rat brain ischemia model [174] and decreased lipid peroxidation to basal levels suggesting SAC beneficial effects in brain ischemia and that the major protective mechanism may be the inhibition of free radical-mediated lipid peroxidation [175].

## 9. Conclusions

**Figure 4:**
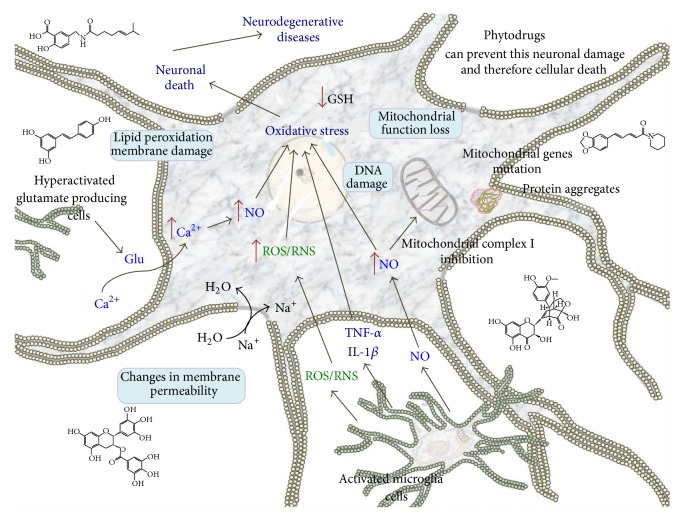
Main neuronal death pathways caused by oxidative stress. Oxidative stress can lead to neuronal death via several mechanisms such as mitochondrial dysfunction, DNA damage, membrane permeability loss, protein aggregation, and apoptosis. Phytodrugs, mainly polyphenols and alkaloids, can prevent this neuronal damage and, therefore, cellular death. Thus, these natural compounds can be used in the treatment of ND and also could serve as models for developing new specific drugs against these pathologies.

Neurodegenerative diseases (ND) are chronic and progressive conditions, characterized by neuronal loss secondary to oxidative stress and neuroinflammation (Figure 4). Until now ND have no cure and represent high costs for the health system and patients families. Exploring alternative sources for ND therapy has led to set eyes on herbal medicine since most herbal compounds have antioxidant and anti-inflammatory properties. At present, the use of several plants in the treatment of ND is being supported by numerous scientific investigations (the main effects of herbal plants against ND are listed in Table 1). However, information is still missing on relevant aspects such as metabolism, pharmacokinetics, and bioavailability in the brain as well as any changes that they may have in the CNS. Nevertheless, plant compounds or extracts remain interesting therapeutic candidates for ND management.

## Figures and Tables

**Table 1:** Main biological effects of phytodrugs in neurodegenerative diseases models.

Effect	Plant compound/extract	Model	Disease/condition	Reference
Antiapoptotic and/or cell viability	*Anemopaegma mirandum *extracts.	*In vitro*	Rotenone model in SH-SY5Y cells.	[137]
	*Pueraria thomsonii. *	*In vitro*	6-OHDA model in PC12 cells.	[155]
	*Cistanche salsa. *	*In vivo*	MPP^+^ mice model.	[127]
	*Gastrodia elata *extract.	*In vitro *	MPP^+^ model in SH-SY5Y cells.	[141]
	*Rosmarinus officinalis *extract.	*In vitro *	A*β* model in cortical neurons.	[168]
	*Chrysanthemum morifolium *extract.	*In vitro*	MPP^+^ model in SH-SY5Y cells.	[126]
	*Panax notoginseng.*	*In vitro*	MPTP model in mesencephalic neurons.	[151]
	Piperine (Figure 2(d)).	*In vitro*	Rotenone model in SH-SY5Y cells.	[104]
	*L-theanine, *from green tea (Figure 3(b)).	*In vitro*	H_2_O_2_ model in SH-SY5Y cells.	[16]
	Toki-to, mixed medicinal herbs.	*In vitro* *In vivo*	6-OHDA model in PC12 cells. 6-OHDA rat model.	[158]
	*Nobiletin, *a flavonoid from citrus peels.	*In vitro* *In vivo *	H_2_O_2_ model in PC12 cells. Rat artery occlusion model.	[95]
	*Psoralea corylifolia.*	*In vitro* *In vivo*	MPP^+^ model in CHO cells and SK-N-SH cells. MPTP mice and rat model.	[161]
	*Chrysanthemum morifolium *extract.	*In vitro *	MPP^+^ model in SH-SY5Y cell.	[126]
	*Uncaria rhynchophylla *extract.	*In vitro*	Rotenone model in SH-SY5Y cells.	[146]
	Polyphenolic extract from* Vitis vinifera.*	*In vitro*	Rotenone model in SH-SY5Y cells.	[93]
	*Withania somnifera *extract.	*In vitro* *In vivo*	MPP^+^ model in CHO cells and SK-N-SH cells. MPTP mice and rat model.	[154]
	Paeonol from* Paeonia suffruticosa *or* Paeonia lactiflora.*	*In vitro* *In vivo*	MPP^+^ model in PC12 cells. MPP^+^ mice model.	[14]
	*Ipomoea batatas *PoirCv.	*In vitro*	A*β* model in PC12 cells.	[121]
	Biotransformed blueberry juice by *Serratia vaccinii bacteria.*	*In vitro*	H_2_O_2_ model in neuronal cells.	[61]
	Polyphenolic from cocoa.	*In vivo*	Aged rats.	[76]
	*Salvia miltiorrhiza.*	*In vitro* *In vivo*	Cortical neurons overexpressing APP695. APP/PS1 transgenic mice.	[88]
	*Opuntia dillenii.*	*In vitro* *In vivo*	NMDA model in cortical neurons. Rat artery occlusion model.	[169]
	*Selaginellin *from* Saussurea pulvinata.*	*In vivo*	A*β* mice model.	[63]
	*Mucuna pruriens.*	*In vivo*	APP-SL 7-5 transgenic mice APP695.	[157]
	Urundeuvines A, B, and C chalcones from* Myracrodruon urundeuva.*	*In vivo*	6-OHDA model in mesencephalic cells.	[147]

Cell survival	*Bacopa monnieri *extract.	*In vitro*	A*β* model in cortical neurons.	[113]
	*Dioscorea opposita. *	*In vitro* *In vivo *	H_2_O_2_ or glutamate model in cortical neurons. Scopolamine mice model.	[106]
	*Nobiletin, flavonoid* from citrus peels.	*In vitro* *In vivo *	H_2_O_2_ model in PC12 cells. Rat artery occlusion model.	[95]
	*Opuntia dillenii.*	*In vitro* *In vivo *	NMDA model in cortical neurons. Rat artery occlusion model.	[169]
	*Pelargonidin *(Figure 1(o)).	*In vivo*	Ethylcholine aziridinium ion model (AF64A).	[133]
	*Psoralea corylifolia. *	*In vitro* *In vivo*	MPP^+^ model in CHO cells and SK-N-SH cells. MPTP mice and rat model.	[161]
	*Withania somnifera *extract.	*In vitro* *In vivo*	MPP^+^ model in CHO cells and SK-N-SH cells. MPTP mice and rat model.	[154]
	Paeonol from* Paeonia suffruticosa *or* Paeonia lactiflora.*	*In vitro* *In vivo*	MPP^+^ model in PC12 cells. MPP^+^ mice model.	[14]
	Pedicularioside A from* Buddleia lindleyana.*	*In vivo*	6-OHDA rat model.	[145]
	*Silybum marianum.*	*In vivo*	MPTP mice model.	[172]
	Toki-to, mixed medicinal herbs.	*In vitro* *In vivo*	6-OHDA model in PC12 cells. 6-OHDA rat model.	[158]
	Urundeuvines A, B and C chalcones from* Myracrodruon urundeuva.*	*In vivo *	6-OHDA model in mesencephalic cells.	[147]
	*Salvia miltiorrhiza.*	*In vitro* *In vivo*	Cortical neurons overexpressing APP695. APP/PS1 transgenic mice.	[88]
	*Centella asiatica *extract*. *	*In vivo*	PSAPP mice.	[92]
	*Ipomoea batatas *PoirCv*. *	*In vitro *	A*β* model in PC12 cells.	[121]
	*Mucuna pruriens.*	*In vivo*	APP-SL 7-5 model in transgenic mice APP695.	[157]
	*Valeriana officinalis *extract.	*In vivo*	Tg2576 transgenic mice.	[138]
	*Luteolin.*	*In vitro*	LPS model in mesencephalic neuron-glia and microglia cells.	[143]
	*Panax notoginseng. *	*In vitro*	MPTP model in mesencephalic neuron.	[151]
	*Piperine *(Figure 2(d)).	*In vitro*	Rotenone model in SH-SY5Y cells.	[104]
	*L-theanine, *from green tea (Figure 3(b)).	*In vitro*	H_2_O_2_ model in SH-SY5Y cells.	[16]
	*Tripterygium regelii methanolic *extract.	*In vitro*	6-OHDA model in mesencephalic cells.	[140]

Antioxidant	Biotransformed blueberry juice by *Serratia vaccinii bacteria.*	*In vitro *	H_2_O_2_ model in neuronal cell.	[61]
	*Rosmarinus officinalis.*	*In vitro* *In vivo*	H_2_O_2_ or rotenone model in SH-SY5Y cells. Dieldrin model in SN4741 cells. Aged rats.	[166]
	*Centella asiatica *extract.	*In vivo *	PSAPP mice.	[92]
	*Chrysanthemum morifolium *extract.	*In vitro *	MPP^+^ model in SH-SY5Y cell.	[126]
	*Ipomoea batatas *PoirCv.	*In vitro *	A*β* model in PC12 cells.	[121]
	*Gastrodia elata *extract.	*In vitro *	MPP^+^ model in SH-SY5Y cells.	[141]
	*Nobiletin, *flavonoid from citrus peels.	*In vitro* *In vivo*	H_2_O_2_ model in PC12 cells. Rat artery occlusion model.	[95]
	*Opuntia dillenii.*	*In vitro* *In vivo*	NMDA model in cortical neurons. Rat artery occlusion model.	[169]
	Methanolic extracts from *Salvia species.*	*In vitro*	Glutamate model in PC12 cells.	[62]
	Toki-to, mixed medicinal herbs.	*In vitro* *In vivo*	6-OHDA model in PC12 cells. 6-OHDA rat model.	[158]
	*Tripterygium regelii *methanolic extract.	*In vitro*	6-OHDA model in mesencephalic cells.	[140]
	Vincamine from* Vinca minor *(Figure 2(c)).	*In vivo*	Mice.	[80]
	Paeonol from* Paeonia suffruticosa *or* Paeonia lactiflora.*	*In vitro* *In vivo*	MPP^+^ model in PC12 cells. MPP^+^ mice model.	[14]
	Oxyresveratrol and resveratrol from* Smilacis chinae rhizome *(Figure 1(c)).	*In vivo*	d-galactose mice model.	[171]
	*Rhus verniciflua *extract.	*In vitro*	H_2_O_2_ model in PC12 cells.	[139]
	*Salvia miltiorrhiza.*	*In vitro* *In vivo*	Cortical neurons overexpressing APP695. APP/PS1 transgenic mice.	[88]
	*Bacopa monnieri *extract.	*In vivo*	A*β* model in cortical neurons.	[113]
	*Buddleia lindleyana.*	*In vivo *	6-OHDA rat model.	[145]
	*Pelargonidin *(Figure 1(o)).	*In vivo*	Ethylcholine aziridinium ion model (AF64A).	[133]
	Samjunghwan, multiherbal extract.	*In vivo*	Acute ischemic stroke model.	[107]

Motor/cognitive improvement	*Silybum marianum.*	*In vivo*	MPTP mice model.	[172]
	Methanolic extracts from* species of Salvia.*	*In vitro*	Glutamate model in PC12 cells.	[62]
	Pedicularioside A from* Buddleia lindleyana.*	*In vivo*	6-OHDA rat model.	[145]
	Paeonol from* Paeonia suffruticosa *or* Paeonia lactiflora.*	*In vitro* *In vivo*	MPP^+^ model in PC12 cells. MPP^+^ mice model.	[14]
	Oxyresveratrol & resveratrol from* Smilacis chinae rhizome *(Figure 1(c)).	*In vivo*	d-galactose mice model.	[171]
	*Magnolia officinalis.*	*In vivo*	SAMP8 mice.	[81]
	*Cistanche salsa.*	*In vivo*	MPP^+^ mice model.	[127]
	Polyphenolic compounds extracted from cocoa.	*In vivo *	Aged rats.	[76]
	*Luteolin.*	*In vitro*	LPS model in mesencephalic neuron-glia and microglia cells.	[143]
	*Cassia obtusifolia. *	*In vivo*	Scopolamine model. Transient cerebral hypoperfusion model.	[102]
	*Dioscorea opposita. *	*In vitro* *In vivo*	H_2_O_2_ or glutamate model in cortical neurons. Scopolamine mice model.	[106]
	Korean red ginseng.	*Clinical trials*	AD patients.	[122]
	*Pelargonidin *(Figures 1(d) and 1(e)).	*In vivo*	Ethylcholine aziridinium ion model (AF64A).	[133]
	*Saussurea pulvinata.*	*In vivo*	A*β* mice model.	[63]
	Toki-to, mixed medicinal herbs.	*In vitro* *In vivo*	6-OHDA model in PC12 cells. 6-OHDA rat model.	[158]
	*Valeriana officinalis *extract.	*In vivo*	Tg2576 transgenic mice.	[138]
	*Piperine *(Figure 2(d)).	*In vitro*	Rotenone model in SH-SY5Y cells.	[104]
	*L-theanine, *from green tea (Figure 3(b)).	*In vitro*	H_2_O_2_ model in SH-SY5Y cells.	[16]

Anti-inflammatory	*Zingiberis Rhizoma *hexane extract*. *	*In vitro *	LPS model in BV-2 microglia cells.	[69]
	*Ficus religiosa *leaf*. *	*In vitro *	LPS model in BV-2 microglia cells.	[68]
	*Luteolin.*	*In vitro*	LPS model in mesencephalic neuron-glia and microglia cells.	[143]
	Samjunghwan, multiherbal extract.	*In vivo*	Acute ischemic stroke model.	[107]
	*Saussurea pulvinata.*	*In vivo*	A*β* mice model.	[63]
	*Silybum marianum.*	*In vivo*	MPTP mice model.	[172]
	*Rosmarinus officinalis. *	*In vitro*	H_2_O_2_ or rotenone model in SH-SY5Y cells.	
			Dieldrin model in SN4741 cells.	[166]
		*In vivo*	Aged rats.	
	*Nobiletin*, flavonoid from citrus peels.	*In vitro* *In vivo*	H_2_O_2_ model in PC12 cells. Rat artery occlusion model.	[95]
	*Cassia obtusifolia. *	*In vivo*	Scopolamine model. Transient cerebral hypoperfusion model.	[102]
	Methoxsalen from* Poncirus trifoliate. *	*In vivo*	Trimethyltin mice model.	[15]
	*Pelargonidin *(Figure 1(o)).	*In vivo*	Ethylcholine aziridinium ion model (AF64A).	[133]
	*Tripterygium regelii *methanolic extract.	*In vitro*	6-OHDA model in mesencephalic cells.	[140]

6-OHDA: 6-hydroxydopamine; A*β*: beta-peptide amyloid aggregation; LPS: lipopolysaccharide; MPP^+^:1-methyl-4-phenylpyridinium; MPTP: 1-methyl-4-phenyl-1,2,3,6-tetrahydropyridine; NMDA: *N*-methyl-D-aspartate.

## Abbreviations

AChE: Acetyl cholinesterase
AD: Alzheimer's disease
A*β*: Amyloid beta-peptide
ALS: Amyotrophic lateral sclerosis
Bax: Apoptosis regulator
Bcl-2: B cell lymphoma 2; family of regulator proteins of apoptosis
BDNF: Brain-derived neurotrophic factor
CNS: Central nervous system
CAMP: Cyclic adenosine monophosphate
DNA: Deoxyribonucleic acid
ERK: Extracellular signal-regulated kinase
GSH: Glutathione reduced
H_2_O_2_: Hydrogen peroxide
IL-1*β*: Interleukin 1*β*
IL-6: Interleukin 6
iNOS: Inducible nitric oxide synthase
LPS: Lipopolysaccharide
MAPK: Mitogen-activated protein kinase
MEK: Mitogen/extracellular signal-regulated kinase
MPP^+^: 1-Methyl-4-phenylpyridinium
MPTP: 1-Methyl-4-phenyl-1,2,3,6-tetrahydropyridine
MS: Multiple sclerosis
NADPH: Reduced form of nicotinamide adenine dinucleotide phosphate
NO: Nitric oxide
NMDA: N-Methyl-D-aspartate
ND: Neurodegenerative diseases
NF-*κ*B: Nuclear factor-*κ*B
Nrf2: Nuclear factor (erythroid-derived 2)-like 2
NOS: Nitric oxide synthase
6-OHDA: 6-Hydroxydopamine-intoxicated mice
PC12: Cell line derived from a pheochromocytoma of the rat adrenal medulla
PD: Parkinson's disease
PN: Panax notoginseng
ROS: Reactive oxygen species
SAC: S-Allyl cysteine
Sjh: Sanmjuanhwan
SCR: Smilacis chinae rhizome
SOD1: Superoxide dismutase 1
TKT: Toki To
TNF*α*: Tumor necrosis factor *α*.
